# Hip Fractures in Long-Term Care: Is the Excess Explained by the Age and Gender Distribution of the Residents?

**DOI:** 10.4061/2010/291258

**Published:** 2010-08-24

**Authors:** Richard G. Crilly, David A. Tanner, Marita Kloseck, Bert M. Chesworth

**Affiliations:** ^1^Division of Geriatric Medicine, Parkwood Hospital, 801 Commissioners Road East, London, ON, Canada N6C 5J1; ^2^Health and Rehabilitation Sciences, Faculty of Health Sciences, University of Western Ontario, London, ON, Canada N6A 5B9; ^3^School of Health Studies, Faculty of Health Sciences, University of Western Ontario, London, ON, Canada N6A 5B9; ^4^School of Physical Therapy, Faculty of Health Sciences, University of Western Ontario, London, ON, Canada N6A 5B9

## Abstract

*Introduction*. This study compares hip fracture rates in Long Term Care (LTC) residents with those in the community to determine if their high rate of fracturing reflects the extreme age and predominantly female nature of that population. *Methods*. Hospital discharge data in London Ontario (population 350,000) and Statistics Canada data were used to correct the hip fracture rate in the LTC setting for age and gender. *Results*. The risk of hip fracture is 1.8 times greater in LTC than in the community for people of similar age and gender. The rate in women is 1.5 times higher whereas in men it is 4.3 times higher. In the oldest residents, the risk in men exceeds that of women in LTC. *Conclusion*. The high hip fracture rate in LTC is not just a reflection of the age and predominantly female nature of this population. The oldest men in LTC are a particularly high risk group, deserving more attention.

## 1. Introduction

Hip fractures are a major problem for the elderly, and especially for those confined to long-term care, both nursing homes and residential homes. Those in institutions constitute about 6%-7% of the population of those over 65 years, but this population contributes almost one third of the hip fracture numbers [1, 2]. The residents of long-term care establishments are, however predominantly female and tend to be particularly old, both risk factors for hip fracture. They are also frailer, which is what usually necessitates their institutionalization and makes them more likely to fall. In addition, they may have fragile bones, though whether more fragile than their community-dwelling peers is not known [3]. In a later report, however, Zimmerman and colleagues demonstrated that the predictors of bone density in nursing home patients were the same as for those living in the community, namely, age, gender, race, and weight [4]. Chen et al. [5] confirmed these associations. 

In this study we have compared hip fracture rate in long-term care (LTC) and community-dwelling men and women at different age strata. Subsequently we corrected for age and gender distribution in the LTC setting to see what proportion of the excess rate of hip fracturing the different age and gender balance of the LTC population might account for. Any remaining excess risk would point to an added risk factor, be it endogenous or exogenous to the patient, as important in increasing the hip fracture rate in these people.

## 2. Methods

The study was conducted in London, Ontario, Canada, a city of approximately 350000 residents, using secondary deidentified data. Hospital discharge abstract databases (DADs) are reported to the Canadian Institute for Health Information from all acute care hospitals in Canada (except those in the province of Quebec) to form a National Database. DADs were obtained from the two local acute care hospitals which handle all hip fractures from the city. Data for 5 calendar years, 2002–2006 inclusive, were obtained.

To distinguish between hip fractures occurring in institutionalized versus community-dwelling populations, a prefracture residence classification was created using information from the residential “postal code”, “place of injury” and “institution from” data fields contained in the DADs. A hip fracture was classified as having occurred in an institution if one of the following three criteria was met: (i) the patient's residential postal code matched a specific postal code for an institution (most of the institutions are large enough to have their own specific postal code), (ii) the patient's residential postal code was consistent with, but not specific to, an institution, and the “place of injury” on the DAD was coded for an institution, or (iii) the patient's residential postal code was consistent with but not specific to an institution, and the patient's “institution from” designation in the DAD was unique to a known institution. Residents of both nursing homes and residential homes are included as we could not distinguish between them in the databases. We refer to this collectively as Long-Term Care.

All subjects aged 65 years or over, who were residents of London, were included in the data set. Patients with subcapital (S72.0-S72.091) or intertrochanteric (S72.1-S72.191) fractures were identified from the data field containing the most responsible diagnosis for the length of stay indicator. Patients coded as having a fracture of the femur other than at the subcapital or intertrochanteric sites, those with identified malignant neoplasms (C00-D09, *n* = 71) or motor vehicle accident-related fractures (V01-V99, *n* = 48) were excluded [6]. Those with more than one admission within a calendar year for hip fracture were only counted once to exclude those with more than one admission for the same fracture or transfer between hospitals. A few (*n* =12) with missing postal codes were excluded. The final number for analysis was 1209 (902 women and 307 men).

The number of London residents for the city of London was obtained from Statistics Canada, Census of Canada data for the years 2001 and 2006 categorized according to census tract. Given that intercensal estimates are not generated at this level of aggregation, population estimates for each year were obtained by linearly interpolating between the two censuses. This assumes a linear change in population numbers and distribution between the two census times. The population count for the middle of the study period (2004) was used in all analyses that amalgamated the study years.

The number of institutionalized people (*n* = 3199) was available from the local agency responsible for admissions to LTC. The age and gender distribution was calculated by a sample survey of 6 LTC homes housing 1448 residents, representing a 45% sample of all LTC residents in the city.

For the institutionalized population the numbers in three age strata 65–74, 75–84, and 85 years and over were calculated for each gender. From the Statistics Canada data the community dwelling population in each age stratum was derived and the rate of hip fracture for each age stratum calculated from this and the DADs. This produced a “standard” rate of fracture for each age stratum which was used to calculate the “expected” or theoretical rate of hip fracture for the institutionalized subjects that would be expected if they had the same rate as those of that age stratum and gender living in the community. A total for each of the actual fracture counts and the theoretical counts could then be summed to provide a comparison of the two counts for the total institutionalized population. This was done for each gender separately and both combined.

Although this is similar to the methods used in other studies, for example, that employed by Guilley et al. [7], there is a difference between the two populations that should be noted. The institutionalized population is essentially stabilized by the beds available in the system. Beds which are vacated, usually in the Canadian system by the demise of the resident, are filled by a new admission which may or may not be of the same gender and in the same age stratum. It is likely, however, that overall, the population is reasonably stable regarding gender and age distribution, in the absence of any major extraneous influences. In the community, people who die are only replaced by younger people aging into the lower stratum. But once again, in the absence of major demographic trends, such as the arrival of the baby boomers, the population will remain reasonably stable in terms of age and gender distribution. There will be some immigration into and emigration from the region of interest somewhat similar to the discharge and admission processes of the institution and which cannot be accounted for. 

Furthermore in the method used whereby secondary data analysis of 5 consecutive years of administrative data are studied, the institution dwellers are not a true cohort as the turnover is higher, and the composition of the cohorts, in terms of the individual members of the study group year by year, will change more than the community dwellers. This turnover may actually be part of the cause of an excess rate of hip fractures as people newly admitted are known to be at higher risk of hip fracture [8]. Accordingly the definition of the rate of fracture presents some difficulty but for simplicity sake we have chosen to call it the rate per person-year but bearing in mind the above provisos. 

Data analysis was performed using SPSS 18, and confidence intervals for ratios followed the method of Altman et al. [9].

## 3. Results

**Figure 1:**
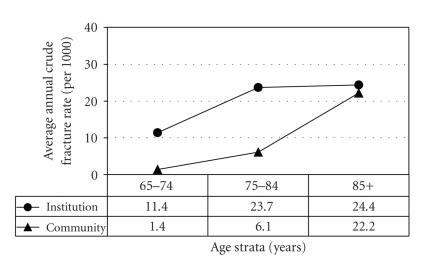
Average annual crude hip fracture rates (person-years) in women by age strata and pre-fracture residence (2002–2006).

**Figure 2:**
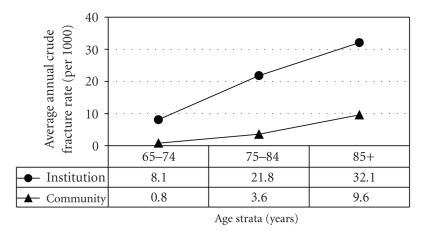
Average annual crude hip fracture rates (person-years) in men by age strata and pre-fracture residence (2002–2006).

Overall 31% of all hip fractures in the city occurred in institutionalized residents. Figures 1 and 2 compare the crude rates of fracture in community and institutional dwelling subjects for the three age strata and the two genders. In the community, the expected rapid rise with age is seen, with women higher than men for all age strata. For men in institutions the fracture rate increases with age and is much higher than that seen in the community men at all age strata. For women the pattern is somewhat different, with the rate tending to plateau and the rate in the oldest band being very similar for women living in the community and those in the institutions. The men in institutions at the two younger age bands have a fracture rate similar to those of women of the same ages whereas the oldest men in institutions actually have a higher rate than the oldest women in institutions (*P* < .05).  Table 1 shows that the mean age of the patients in the various age bands was very similar for community and LTC dwelling patients.

Table 2 shows the actual numbers of hip fractures for men, women, and both together for those living in institutions and the theoretical, or “expected”, number that would be seen if their rate was the same as those of similar age and same gender living in the community, that is, standardized to the community population. The theoretical number of fractures is calculated by multiplying the total institutional population of that age and gender by the rate of fracturing shown by the community dwellers of same age stratum and gender. The ratio of actual over theoretical is shown. For the total of all institutional subjects the rate of fracturing is 1.8 (95% CI 1.6–2.0) times higher than expected, but it is only 1.5 (95% CI 1.3–1.7) times higher for women while being 4.3 (95% CI 3.7–5.6) times higher for men (*P* < .05 for men versus women). Overall, 207 of the 373 institution-based hip fractures or 55% (+/− 2.6 SE) can be explained by the age and gender distribution of the institutional population, but the corresponding percentages explained for the women and men are 66% (+/− 2.8) and 23% (+/− 4.5), respectively. 

## 4. Discussion

Our study suggests that the excess number of hip fractures that are seen in the LTC population is only partly accounted for by the age and gender distribution in this population. Furthermore, a much lower proportion of the fractures in men appears to be attributable to the age distribution than is the case in women. Clearly other factors operate. Hip fractures are a complicated phenomenon produced in part by increasing fragility of the bones with age, an increasing tendency to fall, and a change in the manner of falling in older life, where the fall is more likely to be sideways or backwards [10, 11].The studies of Zimmerman et al.[4] and Chen et al. [5] provided several predictors of hip fracture in the LTC population, namely, age, cognition, gender, race, and weight. The population studied here is almost exclusively of white Caucasian origin and race is not an issue. We did not have access to weight, which may be an important factor as residents of LTC may lose weight. This has been well described in the context of private for-profit nursing homes in the United States, but evidence from a Canadian context is less available [12–14]. Undernutrition, as opposed to actual weight loss, is well described, especially in cognitively impaired residents and, indeed, low body weight is a risk factor for admission to an institution. Where the hip fracture phenomenon fits into this scenario clearly requires more study. Within certain populations it has been shown that weight loss over the years predicts hip fracture occurrence [15]. Cumming [16] was able to explain the difference in hip fracture rate between institution and community on the basis of various potential confounders, such as weight, dementia, diet, activity, and so on, but whether the final common pathway was through thinning of the bones or risk of falling, was not explored. Dementia, for example, is associated with an increased rate of hip fracture, but it is associated especially with an increased rate of falling [17, 18]. Hui et al. [19] demonstrated that for a given bone density there were more fractures with aging, suggesting other factors operate, the obvious one being a tendency to fall more often. Interestingly, none of these studies has corrected for the rate of falling nor may this be the whole answer, as the way of falling may be as important as the frequency.

The possible consequence of the higher turnover in the LTC group needs to be considered. Our hip fracture rates, especially in the older women, are lower than some of the figures reported in the literature. For example, Rapp et al. [8] found a markedly higher rate but their study was a prospective cohort following subjects from the time of admission. Thus this included the early stages of the admission to the home when risk of fracture is highest, due perhaps to disorientation and unfamiliarity with the surroundings, deconditioning from recent illness, and so on, but even allowing for that our rates are low. For example, Chen et al. [5] found an overall rate of 40 per 1000 person years in a prospective study of subjects already residing in LTC. Whether the difference is due to patient factors such as different levels of mobility, or system characteristics, such as admission criteria or programming is not clear. Our study, for example, included both nursing and residential home subjects. 

One might have predicted that the need to be admitted to long-term care would have had a leveling effect on the rate of fracturing as individuals need to have reached a threshold level of dysfunction, and perhaps frailty, in order to be admitted. To some degree this does happen as the relative increase in risk is much higher for the younger strata compared to those in the community (Table 2). However, the observation that for the most part absolute risk is higher for those who are in an institution and are in the most advanced age category suggests that even in older patients, for whom institutionalization seems necessary, increasing frailty is seen with increasing age. 

These results show some interesting differences and interesting similarities between men and women. The markedly higher hip fracture rate in institutionalized men of all ages and institutionalized women in the two younger age strata, compared to those in the community, is notable. The anomaly seems to be the similar rate of fracturing in the oldest women whether in the community or LTC. Possibly, the reasons the oldest women are admitted to LTC are not related to the risk of falling, this therefore remaining the same for both community and LTC dwellers from that stratum. This does not, however, intuitively seem correct. It is known that falling in the LTC population is much more prevalent than in the community but whether this is correct for the oldest female group is unknown. The particularly high fracture rate in men is, perhaps, more readily understood. As more very old men have surviving spouses than do old women, this may enable them to be cared for in the community until they are particularly frail and/or ill, and institutionalization is required. Thus the oldest men may be frailer than their female counterparts. However, this is speculative and the explanation is by no means clear.

The situation in the men, who are presumably admitted to the LTC sector for reasons other than osteoporosis, suggests that the high rate in LTC is not just bone-related and, compared to the oldest men, the lower rate in the oldest institutionalized women, who might be expected to have the most osteoporosis, supports a different etiology. 

Our study has some obvious shortcomings, the main one being an absence of information regarding some of the risk factors, such as weight and cognitive status, in the subjects in the two settings. Likewise, comparative bone density studies also need to be done. Our study does, however, include all hip fractures over a 5-year period so should it be free of selection biases. There is some urgency to sorting out the cause of the excess fracture rate in the LTC setting, where it may be easy to assume that the underlying problem is osteoporosis, leading to the use of osteoporosis medications in this setting despite absence of efficacy for this population [20]. As Van Den Kroonenberg et al. [21] have shown, a fall onto the greater trochanter will generate enough force to break most if not all hip bones, and the real problem of hip fractures in LTC may well be the much more difficult one of falling and the way of falling, with osteoporosis simply determining where the hip will break, with those with thin bones more likely to suffer an intertrochanteric fracture than a subcapital fracture [22].

## Figures and Tables

**Table 1:** Mean age (SD) and hip fracture number of the different age bands.

	Female				Male			
	Community		Institution		Community		Institution	
65–74	70.4 (2.67)	84	71.3 (2.26)	10	70.3 (2.96)	41	68.8 (3.03)	5
75–84	80.4 (2.75)	282	81.1 (2.49)	97	80.5 (2.72)	112	81.7 (2.30)	36
85+	89.6 (3.49)	253	90.1 (3.86)	176	88.9 (3.26)	64	89.6 (2.58)	49
All	82.8 (7.19)	619	86.3 (6.16)	283	81.1 (7.09)	217	84.9 (5.95)	90

**Table 2:** Actual and theoretical incidence of hip fractures in non-community settings by sex and age in the city of London, ON, 2002–2006.

	Institution-based hip fractures			
Sex	Age strata (years)	Actual count	Theoretical count^a^	Ratio^b^
Women	65–74	10	1.21	8.3
	75–84	97	24.97	3.9
	85+	176	160.12	1.1
	All Ages	283	186.30	1.5

Men	65–74	5	0.51	9.9
	75–84	36	5.97	6.0
	85+	49	14.59	3.4
	All Ages	90	21.07	4.3

All	65–74	15	1.72	8.7
	75–84	133	30.94	4.3
	85+	225	174.71	1.3
	All Ages	373	207.37	1.8

*Note*: fracture events ≥65 years of age.

^a^theoretical count calculated as a product of 5 year percent fracture rate of community dwellers and the relevant institutionalized population.

^b^(actual count ÷ theoretical count).
